# Ligneous conjunctivitis presenting after strabismus surgery in the eighth decade of life

**DOI:** 10.1016/j.ajoc.2026.102567

**Published:** 2026-03-17

**Authors:** Alyssa A. Godfrey, Melike Pekmezci, Gerami D. Seitzman, Alejandra G. de Alba Campomanes

**Affiliations:** a Drexel University College of Medicine, 60 N. 36th Street, Philadelphia, PA, 19104, United States; b Department of Ophthalmology, University of California, 490 Illinois St, San Francisco, CA, 94158, United States; c Department of Pathology, University of California, 505 Parnassus Ave, San Francisco, CA, 94143, United States

**Keywords:** Ligneous conjunctivitis, Plasminogen deficiency, Strabismus surgery

## Abstract

**Purpose:**

We present an atypical case of a 71-year-old male with recurrent ligneous conjunctivitis following bilateral rectus muscle recession for strabismus.

**Observations:**

Ligneous conjunctivitis is a rare, chronic form of pseudomembranous conjunctivitis most often seen in infants and children. The condition is typically caused by autosomal recessive variants in the *PLG* gene leading to plasminogen deficiency, resulting in recurrent fibrin-rich lesions on the conjunctiva due to impaired fibrinolysis. Histopathology revealed fibrin-rich material consistent with ligneous conjunctivitis. Serum plasminogen activity and antigen levels were markedly reduced. Whole exome sequencing (WES) identified compound heterozygous variants in the *PLG* gene, confirming plasminogen deficiency. Initial surgical excision resulted in lesion recurrence; however, treatment with subconjunctival fresh frozen plasma (FFP) injections and topical FFP eye drops led to sustained lesion resolution.

**Conclusion:**

This case highlights the late onset of ligneous conjunctivitis in an older adult, likely triggered by surgical trauma. It underscores the importance of considering plasminogen deficiency in recurrent conjunctival lesions, even in older patients.

## 1 Background

Ligneous conjunctivitis is a rare, chronic form of pseudomembranous conjunctivitis that classically presents in infants and children with slight predominance in females.^1^, ^2^, ^3^ It is the most common clinical manifestation of type I plasminogen deficiency, affecting 80% of individuals with the condition. The disorder is usually inherited in an autosomal recessive pattern, resulting from homozygous or compound heterozygous variants in the *PLG* gene.^1^^,^^4^, ^5^, ^6^ These variants lead to plasminogen deficiency, impairing fibrinolysis and promoting the development of fibrin-rich, wood-like pseudomembranous lesions of the conjunctiva.^3^^,^^7^ Pseudomembrane formation may be triggered by trauma, surgery, or infection, and can result in secondary vision loss if left untreated.^3^^,^^8^

## 2 Case description

**Fig. 1:**
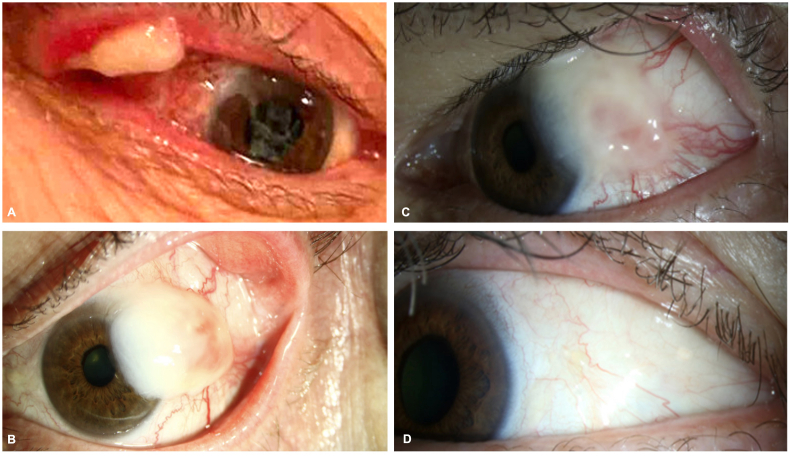
External photographs demonstrating the evolution of conjunctival ligneous lesions A) Right upper palpebral lesion. B) Bulbar conjunctival lesion after strabismus surgery. C) Recurrence of a pseudomembranous lesion after surgical removal of lesion represented in 1B. D) Sustained resolution after 4 months.

A 71-year-old male with a history of CNS follicular lymphoma with posterior fossa involvement presented with nystagmus and exotropia with a chin-down compensatory position for oscillopsia relief. He underwent bilateral superior and lateral rectus recessions. Three weeks after surgery, the patient developed a lesion of the right superior palpebral conjunctiva (self-photographed and represented in Fig. 1A). This lesion was removed by an outside doctor, recurred once, and then spontaneously sloughed without further recurrence. The left conjunctiva then developed a pedunculated wood-like lesion (Fig. 1B) which was surgically removed.

**Fig. 2:**
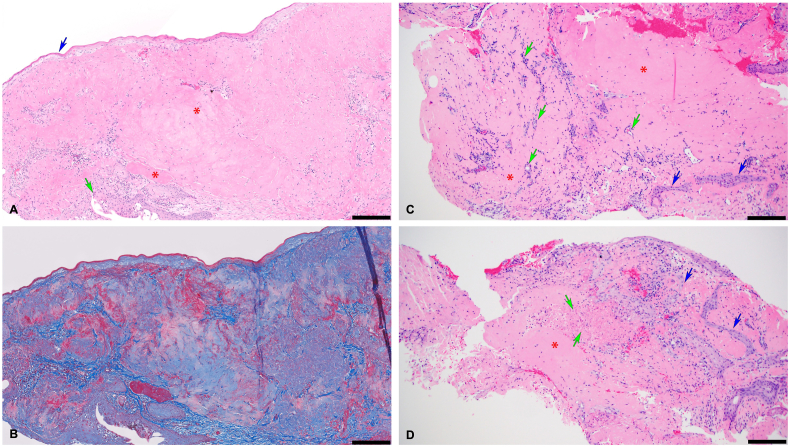
Histologic features of right and left conjunctival lesions of ligneous conjunctivitis. A) Hematoxylin and eosin (H&E)-stained section of the right conjunctival lesion shows eosinophilic glassy material (*) expanding the substantia propria. Conjunctival epithelium is involved by acute inflammation (green arrow) and focally shows parakeratosis and epidermoid metaplasia (blue arrow). B) Masson trichrome-stained section highlights marbled blue and magenta staining corresponding to collagen and fibrin, respectively. C) H&E-stained section of the left conjunctival lesion shows brightly eosinophilic fibrin (*) associated with numerous small vessels (green arrows) and entrapped, invaginations of the surface epithelium (blue arrows). D) H&E-stained section of the left conjunctival lesion shows fibrin (*) entrapping invaginations of the surface epithelium (blue arrows) as well as debris of necrotic epithelial cells (green arrows). (For interpretation of the references to colour in this figure legend, the reader is referred to the Web version of this article.)

Histopathological evaluation of bilateral conjunctival lesions revealed inflamed conjunctiva with reactive epidermoid metaplasia and focal necrosis (Fig. 2). The substantia propria was expanded with glassy, eosinophilic material consistent with fibrin, with associated reactive vessels of granulation tissue and occasional entrapped epithelial cells. Masson trichrome stain highlighted the fibrin, and Congo Red stain was negative, arguing against amyloid deposition. Overall features were those of ligneous conjunctivitis.

Plasminogen activity in serum was 32% (normal: 65-176%) and plasminogen antigen was 1 mg/dL (normal: 8-14 mg/dL). Whole exome sequencing (WES) was performed at the UCSF Clinical Genomic Medicine Laboratory, a CLIA-certified laboratory with a laboratory developed assay, using the patient's saliva sample. A damaging missense variant c.112A > G (p.Lys38Glu) and a truncating frameshift variant c.921dupT (p.Asn308fs) in the *PLG* gene were identified by WES, which are classified as likely pathogenic and pathogenic, respectively, according to the American College of Medical Genetics and Genomics (ACMG) and the Association for Molecular Pathology (AMP) consensus guidelines [ref PMID 25741868]. Given the clinical presentation, these variants are likely *in trans* causing biallelic inactivation; however, these variants are too far apart to be phased on this assay, and segregation analysis was not performed to determine whether they are located on the same or opposite alleles.

Shortly after excision, the lesion returned, this time with a more pseudomembranous presentation (Fig. 1C). The recurrent lesion was excised with adjuvant subconjunctival fresh frozen plasma (FFP) injections and FFP eye drops. This led to complete resolution with no further recurrences (Fig. 1D). Notably, after informed consent, bloodborne pathogen screening, and confirmation of normal levels of serum plasminogen, the FFP was obtained from the patient's wife.

## 3 Discussion

This is an atypical case of an older adult first presenting with signs of ligneous conjunctivitis in the eighth decade of life, after strabismus surgery. This condition usually presents in the second decade of life but has been reported to occur at any age (range of 0-85 years).^3^ Though strabismus surgery has been associated with inciting ligneous conjunctivitis, the previously reported case occurred in a patient at age 3.^9^ Other ophthalmic procedures have been reported in association with the new development of ligneous conjunctivitis, including excision of a pterygium or pinguecula, and cataract surgery.^10^, ^11^, ^12^, ^13^ Additional triggers include ocular trauma or infection^14^^,^^15^ in predisposed individuals, and secondary to medication use^16^^,^^17^ in individuals with no genetic predisposition. One case of a 67-year-old male patient presenting with recurrent lesions after cataract surgery has been reported and was similarly diagnosed with ligneous conjunctivitis based on low plasminogen concentration and plasminogen activity.^12^ It is likely that the delayed presentation of symptoms in our patient is due to his underlying genetic predisposition to plasminogen deficiency, which remained asymptomatic until triggered by surgical intervention. Curiously, our patient did not exhibit signs of disease after undergoing cataract surgery less than a year prior to strabismus surgery. This may be due to the less invasive nature of cataract surgery, which likely caused minimal trauma to the conjunctiva. The palpebral conjunctiva is more commonly involved and lesions on the bulbar conjunctiva are rare;^3^^,^^7^ however, in this case, the insult to the bulbar conjunctiva during strabismus surgery likely triggered the development of the lesions.

Diagnosis of ligneous conjunctivitis is made by clinical findings, plasminogen antigen serum levels and/or plasminogen activity, plasminogen gene analysis, and histopathological findings. Our diagnosis of ligneous conjunctivitis was supported by the patient's low serum plasminogen level and confirmed by two heterozygous variants of the *PLG* gene on WES. Although the identification of *PLG* variants supports the diagnosis of plasminogen deficiency, it is possible that a history of lymphoproliferative disease, chemotherapy, or surgical stress may have acted as precipitating factors in a previously subclinical susceptible state and could explain the unusual late presentation in this patient.^18^

The p.Lys38Glu (p.K38E) missense variant (formerly known as p.K19E) has been reported multiple times in ClinVar (Variant ID:13583) as pathogenic or likely pathogenic by multiple submitters, and is one of the most commonly reported homozygous or compound heterozygous variants in individuals with hypoplasminogenemia. Functional studies have shown that this variant causes decreases in serum plasminogen antigen and activity. However, this variant has a 0.6408% maximum allele frequency in control populations in gnomAD, and computational algorithms produce conflicting evidence regarding predicted functional impact (REVEL score: 0.642, CADD: 17.8, PolyPhen (max): 0.661, phyloP: 1.11). Therefore, this variant has been classified as likely pathogenic according to the ACMG/AMP consensus guidelines [ref PMID 25741868]. Schuster et al. suggest that this *PLG* variant has generally resulted in a higher residual *PLG* antigen and activity and thus a milder clinical course compared to other variants in the plasminogen gene, which may account for the patient's subclinical disease until a triggering event.^5^

To the best of our knowledge, the truncating frameshift variant c.921dupT (p.Asn308fs) has neither been previously published, nor is it reported in databases including ClinVar or The Human Gene Mutation Database (HGMD). This variant is not reported in the 1000 Genomes, ExAC, or gnomAD (normal) population sequencing projects. However, other loss-of-function and truncating variants in *PLG,* including ones downstream of this variant, have been previously reported to be associated with disease.^5^ No functional studies specifically evaluating this frameshift variant have been reported; however, in silico computational algorithms predict deleterious effects (CADD: 40, phyloP: 8.90). Given that this frameshift variant is a truncating variant in a gene where loss of function is a known mechanism of disease (PVS1), is absent from population databases (PM2), is predicted to have a deleterious effect on computational analysis (PP3), and the patient's phenotype is highly specific for the disease (PP4), it has been classified as pathogenic according to the ACMG/AMP consensus guidelines [ref PMID 25741868]. Further research is required to determine how specific *PLG* variants influence the clinical course of the disease.

No standard guidelines exist for treatment of ligneous conjunctivitis. Surgical excision can provide temporary relief of symptoms but is likely to trigger recurrence of lesions. Topical and systemic therapies include plasminogen,^8^^,^^19^, ^20^, ^21^, ^22^, ^23^ fresh frozen plasma,^24^, ^25^, ^26^, ^27^, ^28^, ^29^, ^30^, ^31^ and heparin,^24^^,^^26^^,^^27^^,^^29^^,^^32^, ^33^, ^34^ in tandem with antibiotics,^14^ oral contraceptives,^35^ corticosteroids,^36^ and immunosuppressants.^33^^,^^37^, ^38^, ^39^ After establishing the diagnosis, our patient showed complete resolution of symptoms with administration of subconjunctival and topical fresh frozen plasma and prednisone. Because this patient presented with isolated conjunctival ligneous disease and, at the time of treatment, had no clinical findings suggestive of systemic involvement, a localized (FFP) treatment approach was considered reasonable. Since the care of this patient, intravenous human plasminogen has become available as a systemic therapy for type 1 plasminogen deficiency (PMID 39969447). In cases of recurrent ocular disease or the development of extra-ocular manifestations, systemic plasminogen replacement would now be an important treatment consideration.

Type 1 plasminogen deficiency is a systemic disease. Extraocular lesions in other mucosal sites may occur alongside ligneous conjunctivitis.^3^^,^^7^ Among patients with ligneous conjunctivitis, ligneous periodontitis is the most common site of extraocular lesions, affecting as many as one-third of patients with the condition,^1^^,^^40^, ^41^, ^42^ followed by involvement of the respiratory tract,^43^, ^44^, ^45^ the middle ear,^46^^,^^47^ the female genital tract,^48^, ^49^, ^50^ the gastrointestinal tract, and kidneys. Our patient self-endorsed having had intermittent mouth ulcerations and nondescript skin lesions; however, none were present during his presentation to the eye clinic for our review, nor were they evaluated by other specialties. It is unclear if these reported lesions were related to his diagnosis of ligneous conjunctivitis and no further systemic or dermatologic investigations were pursued.

## 4 Conclusion

Ligneous conjunctivitis is a rare, chronic form of conjunctivitis associated with plasminogen deficiency, characterized both by early pseudomembranous lesions and later by fibrin-rich, woody-like lesions on the conjunctiva. Our case of a 71-year-old male with recurrent ligneous conjunctivitis after strabismus surgery highlights the wide-ranging presentations of this condition.

## CRediT authorship contribution statement

**Alyssa A. Godfrey:** Writing – original draft, Data curation. **Melike Pekmezci:** Writing – review & editing, Investigation. **Gerami D. Seitzman:** Writing – review & editing, Resources, Investigation, Conceptualization. **Alejandra G. de Alba Campomanes:** Writing – review & editing, Supervision, Investigation, Conceptualization.

## Patient consent

Written consent to publish this case has not been obtained. This report does not contain any personal identifying information.

## Authorship

All authors attest that they meet the current ICMJE criteria for Authorship.

## Funding

This work was supported in part by EY002162 (Core Grant for Vision Research, from the NIH-NEI), and an Unrestricted Grant from Research to Prevent Blindness.

## Declaration of competing interest

The authors declare the following financial interests/personal relationships which may be considered as potential competing interests: Gerami Seitzman - Kedrion Biopharma.
